# Laboratory characterisation of *Salmonella enterica* serotype Typhi isolates from Zimbabwe, 2009–2017

**DOI:** 10.1186/s12879-019-4114-0

**Published:** 2019-05-31

**Authors:** Tapfumanei Mashe, Muchaneta Gudza-Mugabe, Andrew Tarupiwa, Ellen Munemo, Sekesai Mtapuri-Zinyowera, Shannon L. Smouse, Arvinda Sooka, Babill Stray-Pedersen, Anthony M. Smith, Joshua Mbanga

**Affiliations:** 1 Department of Applied Biology and Biochemistry, National University of Science and Technology, Bulawayo, Zimbabwe; 2 National Microbiology Reference Laboratory, Harare, Zimbabwe; 3 Letten Foundation Research Centre, Harare, Zimbabwe; 4 Division of Women, Rikshospitalet, Oslo University Hospital and Institute of Clinical Medicine, University of Oslo, Oslo, Norway; 5 Centre for Enteric Diseases, National Institute for Communicable Diseases, National Health Laboratory Service, Johannesburg, South Africa; 6 Faculty of Health Sciences, University of the Witwatersrand, Johannesburg, South Africa

**Keywords:** Antimicrobial resistance, Molecular epidemiology, Molecular sub-typing, PFGE, *Salmonella* Typhi, Zimbabwe

## Abstract

**Background:**

Typhoid fever remains a major public health problem in Zimbabwe with recurrent outbreaks reported since 2009. To provide guidance on appropriate treatment choice in order to minimise the morbidity and mortality of typhoid fever and prevent large scale outbreaks, we investigated the antimicrobial susceptibility patterns, prevalence of *Salmonella enterica* serotype Typhi (*S.* Typhi) H58 haplotype and molecular subtypes of *S.* Typhi from outbreak strains isolated from 2009 to 2017 in Zimbabwe and compared these to isolates from neighbouring African countries.

**Methods:**

Antimicrobial susceptibility testing was performed on all isolates using the disk diffusion, and E-Test, and results were interpreted using Clinical and Laboratory Standards Institute (CLSI) guidelines (2017). *S.* Typhi H58 haplotype screening was performed on 161 (58.3%) isolates. Pulsed-field gel electrophoresis (PFGE) was performed on 91 selected isolates across timelines using antibiotic susceptibility results and geographical distribution (2009 to 2016).

**Results:**

Between 2009 and 2017, 16,398 suspected cases and 550 confirmed cases of typhoid fever were notified in Zimbabwe. A total of 276 (44.6%) of the culture-confirmed *S.* Typhi isolates were analysed and 243 isolates (88.0%) were resistant to two or more first line drugs (ciprofloxacin, ampicillin and chloramphenicol) for typhoid. The most common resistance was to ampicillin-chloramphenicol (172 isolates; 62.3%). Increasing ciprofloxacin resistance was observed from 2012 to 2017 (4.2 to 22.0%). Out of 161 screened isolates, 150 (93.2%) were haplotype H58. Twelve PFGE patterns were observed among the 91 isolates analysed, suggesting some diversity exists among strains circulating in Zimbabwe*.* PFGE analysis of 2013, 2014 and 2016 isolates revealed a common strain with an indistinguishable PFGE pattern (100% similarity) and indistinguishable from PFGE patterns previously identified in strains isolated from South Africa, Zambia and Tanzania.

**Conclusions:**

Resistance to first line antimicrobials used for typhoid fever is emerging in Zimbabwe and the multidrug resistant *S*. Typhi H58 haplotype is widespread. A predominant PFGE clone circulating in Zimbabwe, South Africa, Zambia and Tanzania, argues for cross-border cooperation in the control of this disease.

**Electronic supplementary material:**

The online version of this article (10.1186/s12879-019-4114-0) contains supplementary material, which is available to authorized users.

## Background

Typhoid fever is a significant public health problem with annual estimates of 22 million cases and 216,000 deaths worldwide [1], although the global burden is known to be underestimated, especially in developing countries where the majority of cases likely remain undiagnosed [2]. Typhoid fever is caused by *Salmonella enterica* serotype Typhi (*S.* Typhi, a Gram-negative bacterium, transmitted by ingestion of faecally contaminated food or water. Culture from blood or stool remains the gold standard for typhoid diagnosis, but these methods may not be affordable or practical in low-resource settings, where serological methods have historically been used to diagnose typhoid infection. Even when culture is available, these methods can result in low recovery of the organism (40% blood, 37% stool) and are complicated by the use of antibiotics prior to specimen collection [3]. Clinical presentation varies from a mild illness with low grade fever, malaise and dry cough to a severe clinical picture with abdominal discomfort, altered mental status and multiple complications [4]. If not treated, typhoid fever may progress to severe complications like delirium, intestinal haemorrhage, bowel perforation, and death. Humans are the only natural host and reservoir.

Typhoid fever outbreaks have been recorded in central and southern Africa, affecting both children and adults alike, including in the Democratic Republic of Congo [5], Zambia [6] and Zimbabwe [4, 7]. In Zimbabwe, more than 1000 cases of typhoid fever have been reported annually since 2011, demonstrating the endemicity of the disease. In 2009 [8], a typhoid outbreak primarily affecting two densely populated suburbs of Harare, Mabvuku and Tafara was recorded. Poor sanitation and drinking water quality in these areas and other parts of Zimbabwe were the key risk factors for *S.* Typhi transmission and outbreaks [4]. If detected early and treated with appropriate antibiotics the impact of typhoid fever on an individual and the population is greatly minimised. Antimicrobial susceptibility testing of *S.* Typhi is therefore of great importance in ensuring correct treatment regimens and for monitoring the emergence of any drug resistant strains. In Zimbabwe the treatment guidelines recommend the management of typhoid fever using ciprofloxacin and ceftriaxone [4]. An additional concern is the changing patterns of drug susceptibility for circulating strains of Typhi reported worldwide. Murgia et al. [9] reported that haplotype 58 (H58) is associated with multidrug-resistance to first line drugs, and is the most diffused and rapidly expanding among *S*. Typhi population. The H58 haplotype has also been associated with extremely drug resistance (XDR) Typhoid outbreaks in Pakistan [10]. In addition to the H58 haplotype, S. Typhi with extended β -Lactamase has also been reported in Democratic Republic of Congo (DRC) [11] .However in 2016 Murgia et al. [9] reported that haplotype 58 (H58) is associated with multidrug-resistance to these first line drugs, and is the most geographically dispersed and actively spreading *S*. Typhi haplotype. Surveillance of H58 *S*. Typhi in areas endemic for typhoid fever is therefore key in monitoring the development of resistance to first line drugs and the associated treatment choice in order to effectively minimise the associated morbidity and mortality and prevent large scale outbreaks of *S*. Typhi occurring [9].

Laboratory confirmation of enteric pathogens surveillance was established in Zimbabwe in 1995 and typhoid confirmation was limited to a few laboratories used as sentinel sites.

We present a comprehensive analysis of *S*. Typhi in Zimbabwe identified between 2009 and 2017, for antimicrobial resistance, presence of H58 haplotype and molecular epidemiology, including strain relatedness both within Zimbabwe and with strains from neighbouring countries.

## Methods

### Clinical isolates

Typhoid fever is one of the priority diseases in Zimbabwe and immediate notification of a suspected case is required. Samples were collected from individuals suspected of having typhoid fever are tested at regional and district medical centres. All suspected *S*. Typhi positive samples are referred to the National Microbiology Reference Laboratory (NMRL), Harare for confirmation, quality control and strain collection. For the study, all available isolates were selected.

### Re-culture of isolates and antimicrobial susceptibility testing

Frozen isolates were re-cultured and serotyped based on the White-Kaufman-Le Minor standard method [12]. Confirmed *S.* Typhi isolates were screened for antibiotic susceptibility using the Kirby Bauer disc diffusion method and results were interpreted based on the 2017 CLSI guidelines [13]. The following antibiotics were used; ciprofloxacin (5 μg), ceftriaxone (30 μg), chloramphenicol (30 μg), nalidixic acid (30 μg), tetracycline (30 μg) and ampicillin (10 μg) (Oxoid, United Kingdom). Minimum inhibitory concentration (MIC; mg/L) for ceftriaxone, ciprofloxacin, and azithromycin were done using the E-test (bioMérieux, Marcy l’Étoile, France). *Escherichia coli* ATCC 25922 was used as a quality control. Multi-drug resistance (MDR) was defined as acquired non-susceptibility to at least one agent in three or more antimicrobial categories [14].

### Molecular identification of *S*. Typhi

Deoxyribonucleic acid (DNA) was extracted using a standard heat lysis protocol. In brief a half loopful of bacterial culture (approximately 1 cm sweep across the agar culture) was inoculated into 400 μl sterile (Tris- EDTA) TE buffer and boiled for 25 min at 95 °C on a heating block. The solution was allowed to cool on ice or to room temperature. Bacterial cells were spun for 3 min at 9000 rpm. A 20 μl aliquot of supernatant was added to 80 μl of sterile TE buffer. Quantification of crude DNA was performed using a BioDoc analyse (Biometra, Germany). Crude DNA between 5 and 10 ng/ μl was used for polymerase chain reaction (PCR) reactions.

All the 276 isolates were confirmed using Multiplex real-time PCR targeted two genes: a gene unique to *Salmonella enterica* (ttrRSBCA) and a gene unique to *S.* Typhi (STY0201) [15, 16]. The PCR reaction was carried out using a PCR master mix, TaqMan gene expression (ThermoFisher Scientific, Waltham, MA, USA), primers and probe (Table 2) in a 50 μl final volume reaction. The PCR was run and results analyzed using the Applied Biosystems 7500 real time PCR system (Life Technologies, Foster City, CA) with cycling conditions as follows: 50 °C for 2 min (1 cycle), followed by 95 °C for 10 min (1 cycle), followed by 95 °C for 15 s and 60 °C for 1 min (40 cycles).

### *S*. Typhi H58 haplotype screening

Conventional PCR was used to screen 161 isolates for *S.* Typhi H58 haplotype [9] using a PCR master mix, Dream taq (ThermoFisher Scientific, Waltman, MA, USA) reagent in a 10 μl reaction containing 3.68 μl of nuclease free water, 5.0 μl of master mix, 0.16 μl of 0.4 μM of each primer and 1 μl of DNA template. The PCR was run using the Gene Amp PCR system 9700 (Applied Biosystems, USA). A 25 μl reaction cycle was set up as follows: initial denaturation at 95 °C for 2 min, and 30 cycles of denaturation at 95 °C for 30 s, primer annealing at 58 °C for 30s and extension at 72 °C for 30 s, followed by final extension at 72 °C for 7 min. The PCR products were subjected to electrophoresis in a 1% agarose gel and visualized with ethidium bromide staining; results were checked using the Uvipro silver gel viewer (Uvitec, UK).

### Pulsed-field gel electrophoresis

We used the methodology previously described by Smith et al [17] using using a PulseNet protocol [18]. A pulsotype (PT) was defined as a unique electrophoretic banding pattern. Strains with identical restriction profiles (*Xba*I) were assigned as the same subtype.

## Results

**Fig. 1:**
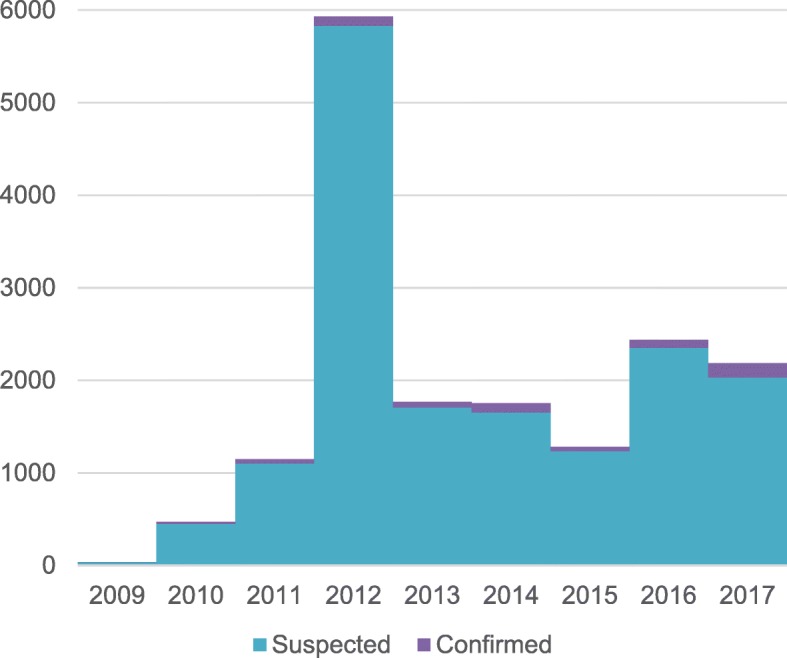
Number of typhoid fever cases notified in Zimbabwe, 2009–2017

Between 2009 and 2017, 16,398 suspected cases and 619 confirmed cases of typhoid fever were notified in Zimbabwe (Fig. 1). A sharp increase was seen from just over 1000 cases being reported in 2011 to nearly 6000 cases in 2012 when a major outbreak was recorded. For the subsequent 5 years between 1300 and 2400 cases were reported annually. The proportion of confirmed cases ranged from 2% in 2012 to 7% in 2017 (Table 1). No isolates were available from 2011 for analysis and it was determined that isolates from 2009 and 2010 would be utilised as the reference strains. Therefore 550 cases were confirmed during the period of interest from 2012 to 2017 and of these 276 isolates (50%) isolated from blood and stool specimens were available in the national *S*. Typhi biobank for analysis (Table 1). PFGE results of isolates from 2009 and 2010 isolates were used as reference for molecular subtyping, as they represent the first recorded typhoid outbreak samples in Zimbabwe.

**Table 1:** Proportion of confirmed cases and retrieved *S*. Typhi isolates analysed, Zimbabwe, 2012–2017

Year	No. suspected cases	No. of confirmed cases	Proportion of all cases confirmed	No. isolates retrieved for analysis	Proportion of all cases with isolates available	Proportion of confirmed cases with isolates available
2012	5829	103	2%	101	2%	98%
2013	1707	61	3%	36	2%	59%
2014	1653	101	6%	24	1%	24%
2015	1236	45	4%	4	0%	9%
2016	2352	85	3%	70	3%	82%
2017	2032	155	7%	41	2%	26%

### Antimicrobial susceptibility assay

**Fig. 2:**
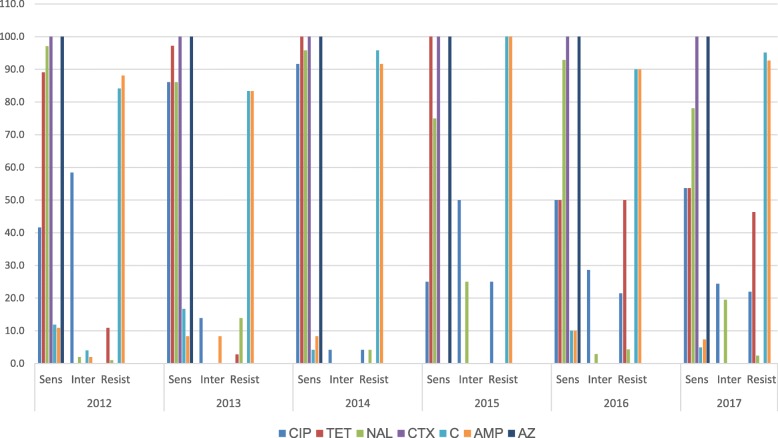
Antimicrobial susceptibility of *S*. Typhi isolates in Zimbabwe (2012–2017). *CIP* Ciprofloxacin, *TET* Tetracyline, *NAL* Nalidixic acid, *CTX* Ceftriaxone, *C* Chloramphenicol, *AMP* Ampicillin, *AZ* Azithromycin, *Sens* Sensitive, *Inter* Intermediate, *Resist* Resistance

A change in antimicrobial susceptibility patterns was observed for *S.* Typhi isolates annually (Fig. 2). Overall trends showed an increase in resistance to ciprofloxacin from 2012 (0%) to 2017 (22%) (Fig. 2). The 25.0% in 2015 was likely due to few samples available for testing. During the same timeframe high intermediate resistance (0.5 mg/L) of ciprofloxacin was also observed (Fig. 2). The ciprofloxacin-resistant isolates had MIC range of 1–2 mg/L. All S. Typhi isolates were sensitive to ceftriaxone (100%) across the six-year period.

An increase in tetracycline resistance was observed from 2012 (11.0%) to 2017 (46.3%), however in 2014 and 2015 isolates were fully susceptible (Fig. 2). The isolates from 2016 and 2017 also had higher levels of resistance to tetracycline. A correlation between ciprofloxacin and tetracycline resistance was observed as depicted by pattern C and D (Table 2). Resistance of isolates to ampicillin ranged between 83.3 to 100% in all the years (Fig. 2) and resistance to chloramphenicol was similarly high at between 83 and 100%. However, all isolates examined through 2017, remained susceptible to ceftriaxone and azithromycin.

**Table 2:** Resistotypes of 276 *S.* Typhi isolates in Zimbabwe (2012–2017)

Pattern	Number of isolates	Percentage	Resistotypes
A	172	62.3	AMP-C
B	1	0.4	CIP-AMP-C
C	20	7.2	CIP-TET-AMP-C
D	1	0.4	CIP-TET-AMP
E	1	0.4	CIP-NAL-AMP-C
F	2	0.7	NAL-AMP
G	6	2.2	NAL-AMP-C
H	3	1.1	TET-C
I	32	11.6	TET-AMP-C
K	3	1.1	TET-AMP
L	2	0.7	TET-NAL-AMP

*CIP* Ciprofloxacin, *TET* Tetracyline, *NAL* Nalidixic acid, *CTX* Ceftriaxone, *C* Chloramphenicol, *AMP* Ampicillin, *AZ* Azithromycin

A total of 11 resistance patterns were observed (Table 2). Pattern A resistance to ampicillin and chloramphenicol was the most common pattern (62.3%) among *S.* Typhi isolates (Table 2). Other prevalent resistotypes included Pattern C (ciprofloxacin-tetracycline-ampicillin-chloramphenicol) and Pattern I (tetracycline-ampicillin-chloraphenicol). A total of 243 *S.* Typhi isolates (88. 0%) were multi drug resistant as they were resistant to two or more drugs (Table 2).

### Haplotype screening

Of the 161 isolates selected based on resistance patterns were screened for H58 haplotype, 150 were positive (93.2%) (data not shown). All the H58 positive isolates were resistant to ampicillin, chloramphenicol and others showed reduced susceptibility to ciprofloxacin. The results of the study shows that H58 associated MDR are widespread among *S.* Typhi isolates in Zimbabwe.

### Molecular subtyping of *S.* Typhi

**Fig. 3:**
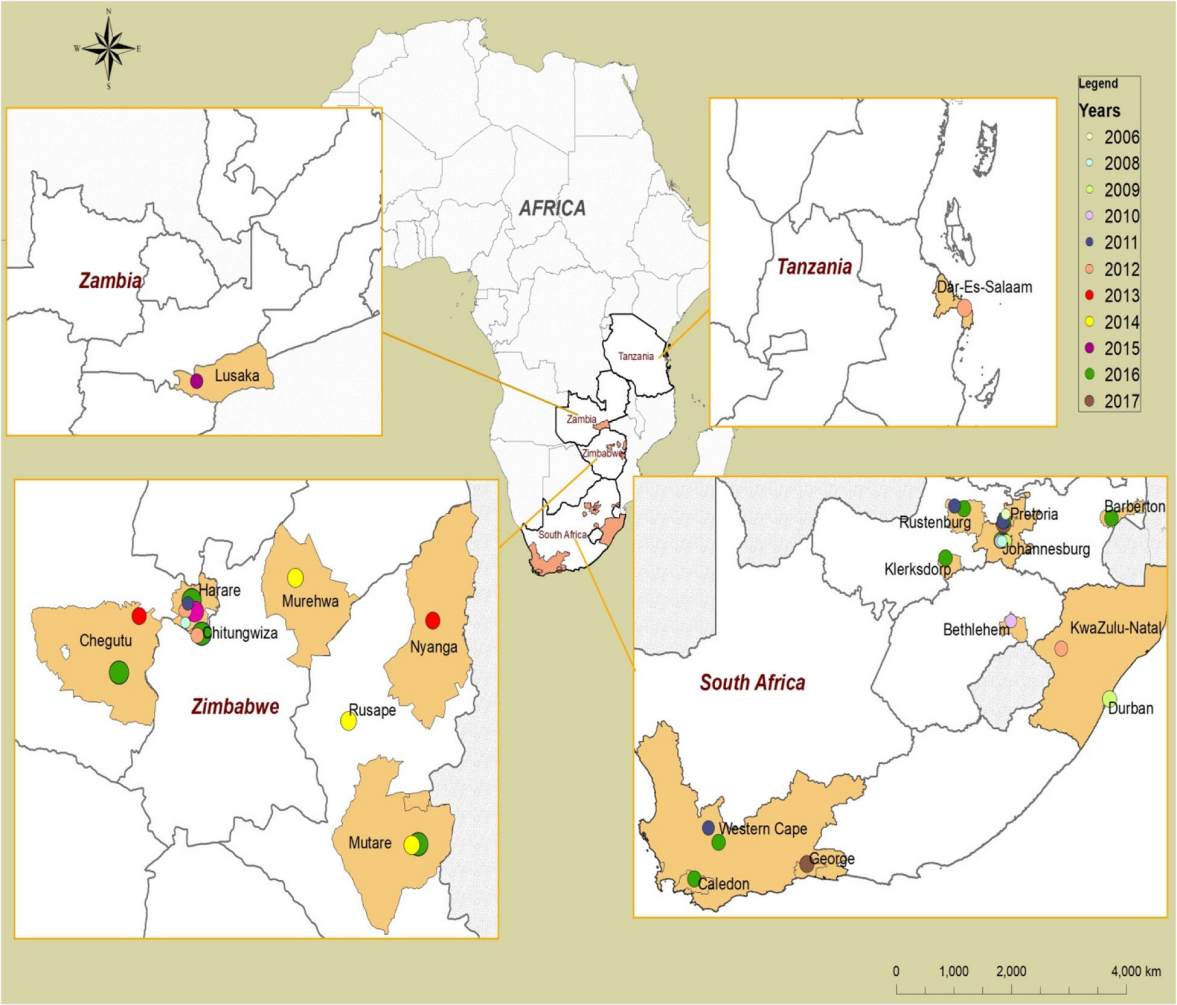
Distribution of *S*. Typhi PFGE clone in South Africa, Tanzania, Zambia and Zimbabwe, 2006–2017

A total of 91 (33%) isolates were selected from the 276 isolates using analytical cross sectional study design to cater for heterogeneous characteristics like year of isolation, antimicrobial susceptibility testing results and geographical area of isolation. The geographic origins of the 91 isolates included Harare, Chegutu, Mutare, Inyanga, Mutawatawa, Rusape, Chitungwiza and Bindura. Dendrogram analysis of PFGE patterns for isolates showed that percentage pattern similarity values ranged between 46 and 100%. The discrimination index was high for PFGE and the technique was able to distinguish between isolates. There was high genetic diversity among *S.* Typhi *isolates* as the 91 isolates were differentiated into a total of 12 PFGE subtypes. The 2009 *S.* Typhi PFGE subtype was indistinguishable (100% similar) from the subtypes of the 2011 isolates and to 68.3% of the 2012 isolates (Additional file 1: Figure S1). The 2013 (8/9), 2014 (6/14) and 2016 (14/20) isolates had subtypes that were 97% similar to the 2009 subtype. A common *S*. Typhi subtype was noted to be circulating in Harare, Mutawatawa, Chitungwiza, Mutare, Rusape and Inyanga. PFGE analysis of the 2012, 2013, 2014 and 2016 subtype revealed an indistinguishable PFGE pattern with the isolates from South Africa (2017), Zambia (2015) and Tanzania (2012) (Fig. 3). It also revealed that the 2009 Mabvuku subtype was 100% similar to the 2006 and 2008 Gauteng, South Africa isolates (Additional file 1: Figure S1). Molecular subtyping of the ciprofloxacin-resistant isolates from different suburbs in Harare, revealed that they all shared a similar subtype.

## Discussion

To provide guidance on appropriate treatment choice in order to minimise the morbidity and mortality associated with typhoid fever and prevent large scale outbreaks a phenotypic and genotypic analysis was conducted on S. Typhi isolates collected from 2012 to 2017. To determine the development of drug resistance to first line antibiotics for typhoid fever and the prevalence of *Salmonella enterica* serotype Typhi (*S.* Typhi) H58 haplotype standardised methodology were performed. *S.* Typhi isolates showed a changing pattern in antimicrobial susceptibility across the years for which isolates were available (2012–2017). Fluoroquinolones such as ciprofloxacin are recommended by WHO [19], as they are reliably effective, inexpensive and well-tolerated drugs for the treatment of typhoid fever [19]. Ciprofloxacin is used as a first line treatment drug for typhoid in Zimbabwe [4]. In this study, an increase in resistance to ciprofloxacin was observed from the 2014 (4.2%) to 2017 (22.0%) isolates (Fig. 2). The ciprofloxacin-resistant isolates were from Harare with Budiriro and Glenview having the highest number in 2016. These ciprofloxacin-resistant isolates have spread to others areas like Mbare, Kambuzuma, Kuwadzana and Hatcliff. Also an increase in intermediate resistance (0.5 mg/L) of ciprofloxacin was recorded from 2014 to 2017 (Fig. 2). Intermediate resistance was observed in 5 isolates from Mutare in 2016. An MIC value 0.5 mg/L was recorded in all isolates showing intermediate resistance meaning ciprofloxacin may be effective at higher doses. Though fluoroquinolone resistance is chromosomally mediated [6], selective pressures exerted by the overuse of these drugs may result in such isolates becoming more common in the future. This may explain the increase in ciprofloxacin resistance in *S.* Typhi isolates in Zimbabwe (especially in Harare) where the antibiotic is used as a broad-spectrum drug to treat many diseases. Resistance and intermediate resistance to ciprofloxacin has been reported from many regions worldwide, including Kenya [20], Cambodia [21], Bangladesh [22] and South Africa [23]. A sharp increase in tetracycline resistance was observed from 2012 (11.0%) to 2017 (46.3%) (Fig. 2). All the tetracycline-resistant*S.* Typhi isolates from 2016 were isolated in Harare. In Zimbabwe, tetracycline is not used as a drug of choice for the treatment of typhoid fever but it is heavily used in the poultry industry and may be indicative of human exposure to residual antibiotics in the food chain. Strains that acquire this type of resistance also become co-resistant to other antibiotics such as Beta-lactams and fluoroquinolones, if resistance is plasmid-borne [24]. A correlation between tetracycline and ciprofloxacin resistance was observed (Table 2). In this study, all the ciprofloxacin resistant strains were susceptible to ceftriaxone and azithromycin (Fig. 2).

All the isolates from 2012 to 2017 were susceptible to ceftriaxone (Fig. 2). Intravenous ceftriaxone is a drug of choice for typhoid treatment in Zimbabwe [4]. In addition, it is used to treat typhoid fever due to resistant bacteria [19]. Resistance to older first-line drugs for *S*. Typhi such as ampicillin and chloramphenicol remained constantly high ranging from 83.3 to 100% (Fig. 2). In a similar study done in India, 75.5% of *S.* Typhi isolates were resistant to amoxicillin [25]. Ampicillin resistance can be used to predict resistance of *S.* Typhi to amoxicillin [13]. Globally, extremely high resistance to ampicillin and chloramphenicol, [5, 25, 26] has motivated for the use of alternate antibiotics for typhoid fever, but our results suggest that increasing ciprofloxacin resistance may soon render this antimicrobial ineffective in typhoid fever control programmes. Our findings warrant an adjustment in typhoid treatment guidelines and a shift towards evidence based management and routine antimicrobial resistance surveillance programs in Zimbabwe.

Multidrug resistant strains are a major therapeutic concern for physicians in developing countries. Contributing factors may include antimicrobial misuse and inappropriate prescribing practices [27] as well as intrinsic plasmid-mediated factors [22, 28, 29]. Eleven multidrug resistance patterns were observed and the most common pattern, resistotype A (resistance to ampicillin-chloramphenicol) was exhibited by 172 (62.3%) isolates (Table 2). The high level of resistance to first-line antimicrobials for treatment of typhoid fever is worrisome, as 243 *S.* Typhi isolates (88.0%) were resistant to two or more antimicrobials and 150 of the 161 tested belonged to the H58 haplotype. Results of the study suggest high prevalence of MDR H58 haplotype in clinical *S.* Typhi isolates in Zimbabwe. According to a study done by Wong et al. [29] 63% of *S*. Typhi isolates belonged to H58 lineage in Eastern and Southern Africa. The H58 lineages I and II were detected in Kenya, Tanzania, Malawi and South Africa [30], neighbouring countries to Zimbabwe.

Outbreaks of MDR *S.* Typhi strains have been reported around the world. In 2011, researchers in Malawi isolated MDR H58- lineage *S*. Typhi in Blantyre, Malawi [31]. Multidrug resistant strains of S. Typhi have been reported from many African countries, including Kenya, Uganda, Tanzania and Ghana [32]. Due to the presence of MDR and quinolone-resistant*S.* Typhi isolates [33], it has been recommended that developing countries should use azithromycin as a first-priority drug.

PFGE analysis was used for molecular subtyping of isolates and to determine relatedness of 91 *S.* Typhi isolates from 2009 to 2016. PFGE is a powerful molecular biology technique which has provided important insights into the epidemiology and population biology of many pathogens in the world [34]. In the present study, 12 PFGE subtypes were shown amongst the 91 isolates. PFGE is regarded as one of the most reliable techniques for discriminating different strains of *S.* Typhi [35, 36]*.* The same subtype observed for the 2009 Mabvuku isolates was consistently seen in South African samples of 2006, 2008, 2009, 2010, 2011, 2012, 2016 and 2012 (Zimbabwe) (Additional file 1: Figure S1, Fig. 3) suggesting that the strain is circulating in Zimbabwe and South Africa. The Mabvuku 2009 subtype was noted to be circulating in Harare (2013; 2016), Mutawatawa (2014), Chitungwiza (2012), Mutare (2016), Rusape (2014) and Inyanga (2013), demonstrating a relationship between isolates across a wide area and timeline. These findings point toward Mabvuku as the source of 2009 typhoid resurgence in Harare, Zimbabwe. Some PFGE subtypes were unique to particular towns such as Masvingo, Mutare and Chegutu.

Resistance traits (e.g. fluoroquinolone resistance) were highly subtype-specific, suggesting predominantly subclonal distribution. Although the proportion of all cases with an available isolate is small due to the sampling process within a country these findings still remain key in advancing our understanding of the genetic structure, ecology, geographic distribution, and emergence of this widely disseminated drug–resistant pathogen, which represents a growing public health threat. It does however point to the need to improve sample collection processes for individuals suspected of having typhoid fever. Our research findings also revealed that there is a common *S*. Typhi strain circulating in Zimbabwe, South Africa, Zambia and Tanzania as evidenced by a common subtype in the isolates (Fig. 3). Imanishi et al. [8] also observed that there was a common subtype circulating in Zimbabwe, Malawi and Tanzania when they analyzed their 2009 and 2011 isolates. Similarities between PFGE subtypes from multiple countries may be the result of population movements in Zimbabwe, Zambia, South Africa and Tanzania where people move easily from one country to another.

## Conclusions

In Zimbabwe there is emerging antimicrobial resistance to first line drugs (ciprofloxacin, amoxicillin and chloramphenicol) used for typhoid treatment and widespread distribution of MDR H58 *S*. Typhi isolates. Treatment recommendations should therefore be based on these laboratory sensitivity results. *S.* Typhi strains in Zimbabwe are presently susceptible to ceftriaxone and azithromycin: use of these drugs for treatment of typhoid fever should be promoted. PFGE results suggest there are 12 strains of *S*. Typhi in circulation in Zimbabwe and that the 2009 Mabvuku strain is still in circulation. A better understanding of the molecular epidemiology of *S.* Typhi in Zimbabwe can greatly contribute to the prevention and control of outbreaks as well as determine cross-border spread by providing the scientific evidence to develop appropriate comprehensive and integrated strategies.

## Additional file

Additional file 1: **Figure S1.** PFGE analysis of the Zimbabwe (2009) with South Africa (2006, 2008, 2009, 2010, 2011 and 2012) *S.* Typhi isolates. (DOCX 187 kb)

## Data Availability

The datasets analyzed during the current study are available from the corresponding author on reasonable request.

## Abbreviations

CLSI: Clinical and Laboratory Standards Institute
DNA: Deoxyribonucleic acid
DRC: Democratic Republic of Congo
MDR: Multi-drug resistance
NMRL: National Microbiology Reference Laboratory
PFGE: Pulsed-field gel electrophoresis
TE: Tris- EDTA
USA: United States of America
WHO: World Health Organization
XDR: Extremely drug resistance
