# Supplementary figures and images for: Laboratory characterisation of Salmonella enterica serotype Typhi isolates from Zimbabwe, 2009–2017
Source: BMC Infect Dis. 2019 May 31;19:487. doi: 10.1186/s12879-019-4114-0 (PMC6544939; doi:10.1186/s12879-019-4114-0)

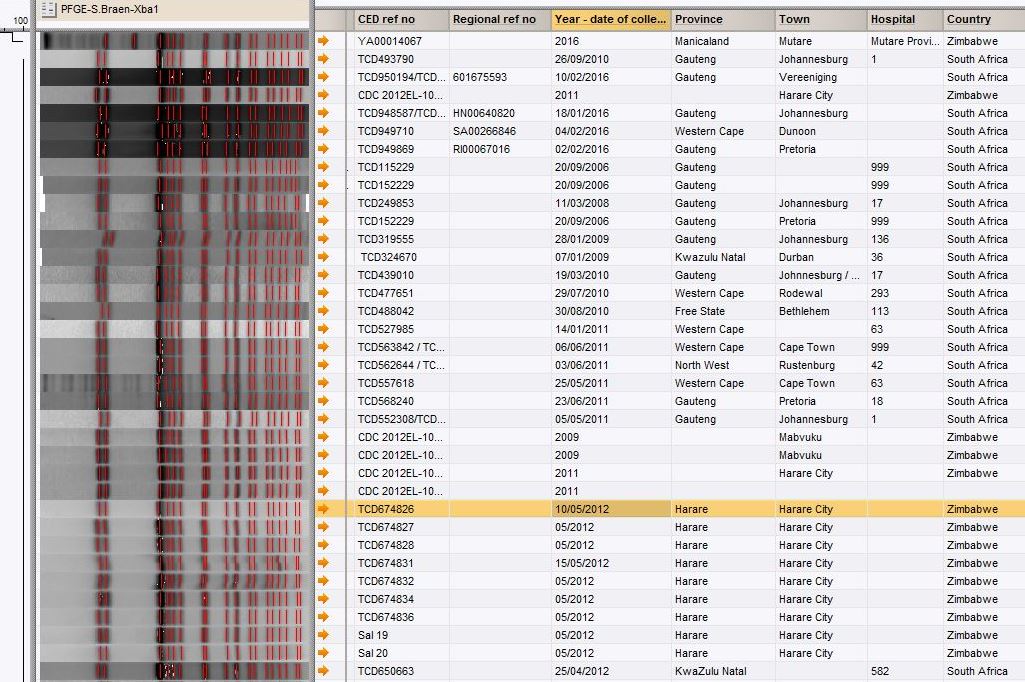


**Additional file 1: Figure S1**: PFGE analysis of the Zimbabwe (2009) with South Africa (2006, 2008, 2009, 2010, 2011, and 2012) *S.* Typhi isolates

Supplement: Supplementary file 1 — Figure S1. PFGE analysis of the Zimbabwe (2009) with South Africa (2006, 2008, 2009, 2010, 2011 and 2012) S. Typhi isolates. (DOCX 187 kb) [file 12879_2019_4114_MOESM1_ESM.docx]
